# Supplementary material for: SPREd: A simulation-supervised neural network tool for gene regulatory network reconstruction
Source: bioRxiv. 2023 Nov 13:2023.11.09.566399. Preprint. [Version 1] doi: 10.1101/2023.11.09.566399 (PMC10680606; doi:10.1101/2023.11.09.566399)
Supplement: Supplement 1 [file media-1.pdf]

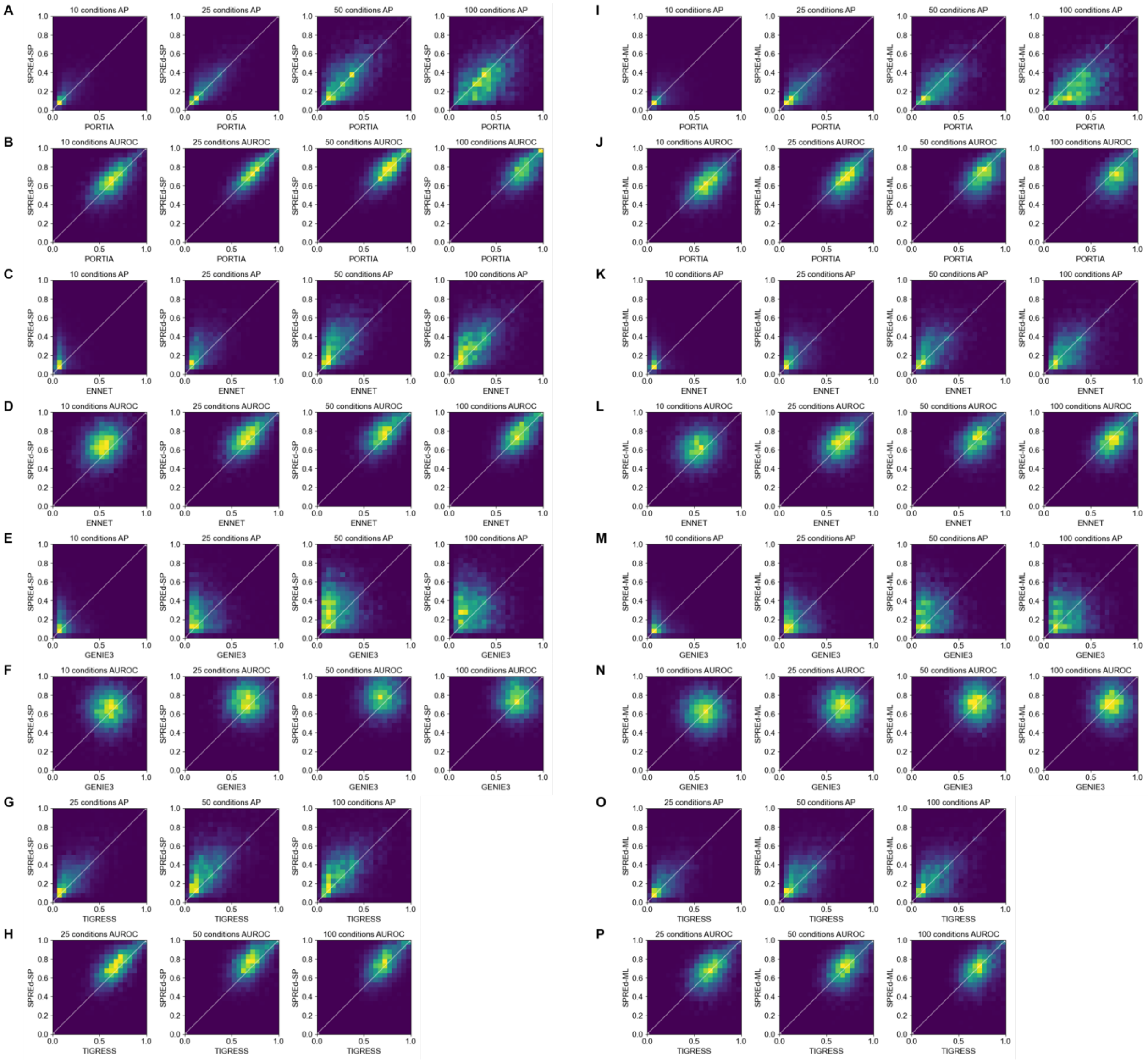

Supplementary figure S1. Direct comparisons of AP (**A**, **C**, **E**, **G**, **I**, **K**, **M**, **O**) and AUROC (**B**, **D**, **F**, **H**, **J**, **L**, **N**, **P**) between SPREd-SP and PORTIA (**A**, **B**), SPREd-SP and and ENNET (**C**, **D**), SPREd-SP and GENIE3 (**E**, **F**), SPREd-SP and TIGRESS (**G**, **H**), SPREd-ML and PORTIA (**I**, **J**), SPREd-ML and ENNET (**K**, **L**), SPREd-ML and GENIE3, or between SPREd-ML and TIGRESS (**O**, **P**).

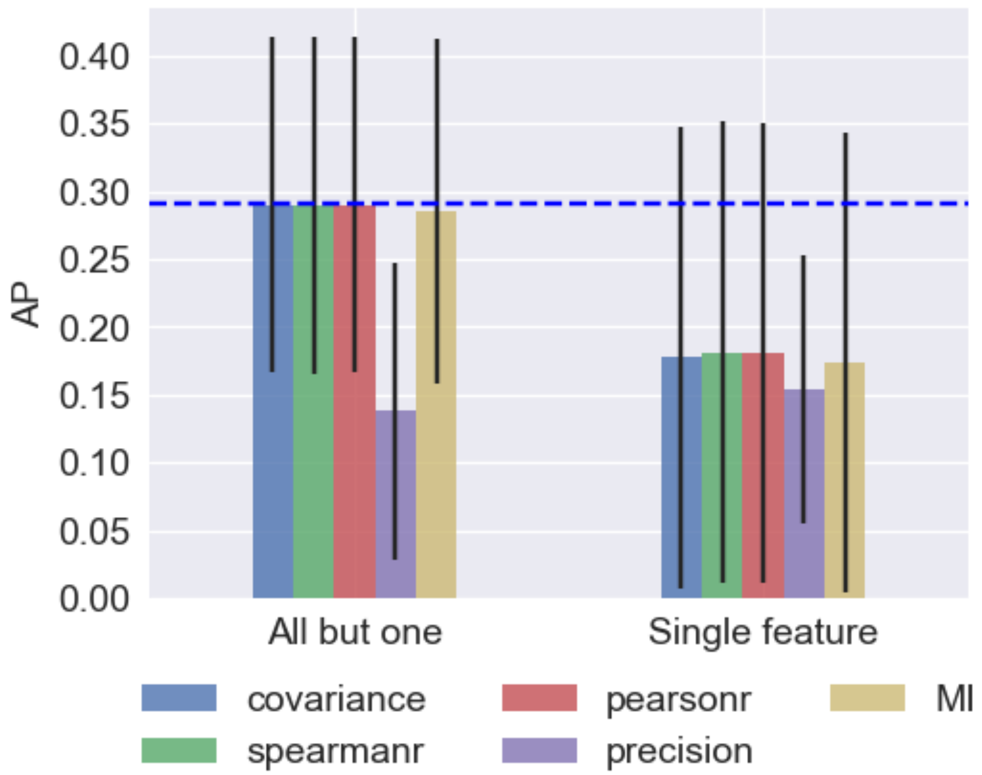

Supplementary figure S2. Average Precision of SPREd-ML when using all but one (left) or only one (right) of the five features describing each TF-gene or TF-TF pair. AP when using all five features is shown in blue dashed line.

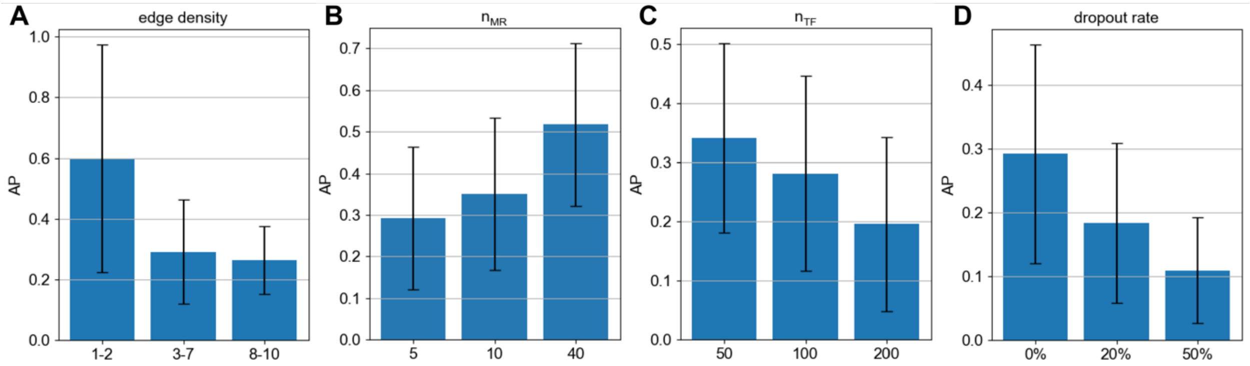

Supplementary figure S3. AP scores of SPREd-ML for varying benchmark parameters. Performance (average precision) of SPREd-ML with varying edge density ($d_{TF \to G}$) of 1-2, 3-7, and 8-10 TFs per target gene (**A**), varying numbers of MRs (n_{MR}) (**B**), varying numbers of TFs (n_{TF}) (**C**), and varying levels of dropout added to the synthetic expression matrix (**D**).

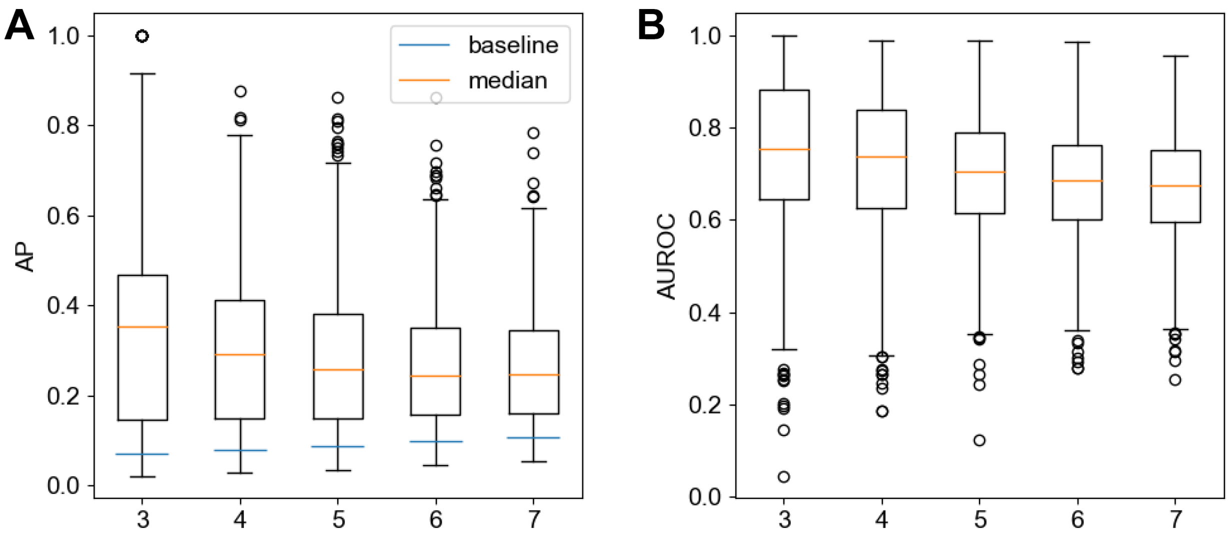

Supplementary figure S4. SPREd performance (with default GRN parameters of $n_{MR} = 5$, $n_{TF} = 100$, $d_{MR \to TF}$ = 2) at different values of edge density $d_{TF \to G}$ = 3, 4, … 7. Average Precision (AP) is shown in panel (A) and AUROC is shown in panel (B). The blue line in (A) shows the random expectation of AP.

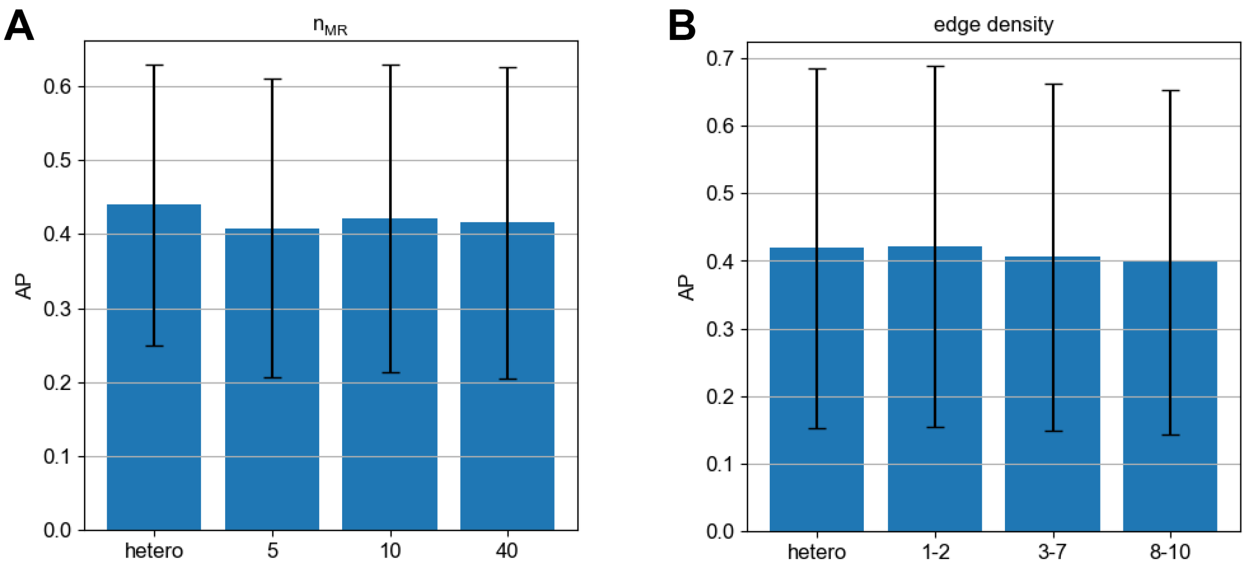

Supplementary figure S5. Performance comparison on heterogeneous benchmarks. (**A**) Performance of SPREd-SP on heterogeneous test sets of different using model weights from training sets with n_{MR} set to 5, 10, and 40 or a mix thereof ("hetero"). (**B**) Performance of SPREd-SP on heterogeneous test sets of varying edge densities using model weights trained on datasets with different edge densities – 1-2, 3-7, 8-10, or the entire range ("hetero").

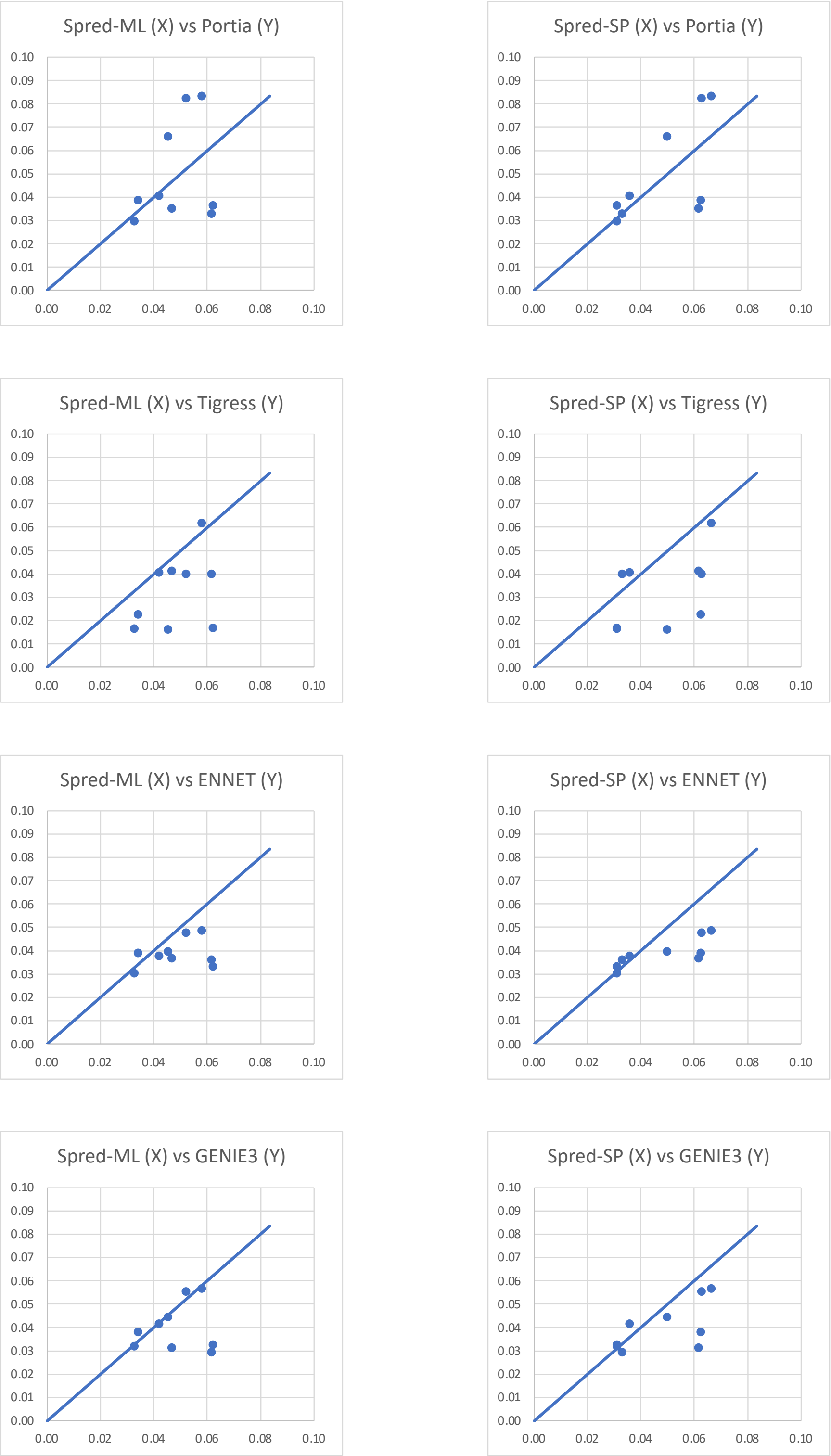

Supplementary figure S6. Head-to-head comparison of SPREd-ML (left) or SPREd-SP (right) versus each comparator (PORTIA, TIGRESS, ENNET, GENIE3) in terms of Average Precision (AP) on each of the nine benchmarks ("tests"). In each panel, the nine points shown represent the tests, X-axis is the AP (averaged over all genes) of a SPREd model and Y-axis is the AP of a comparator.

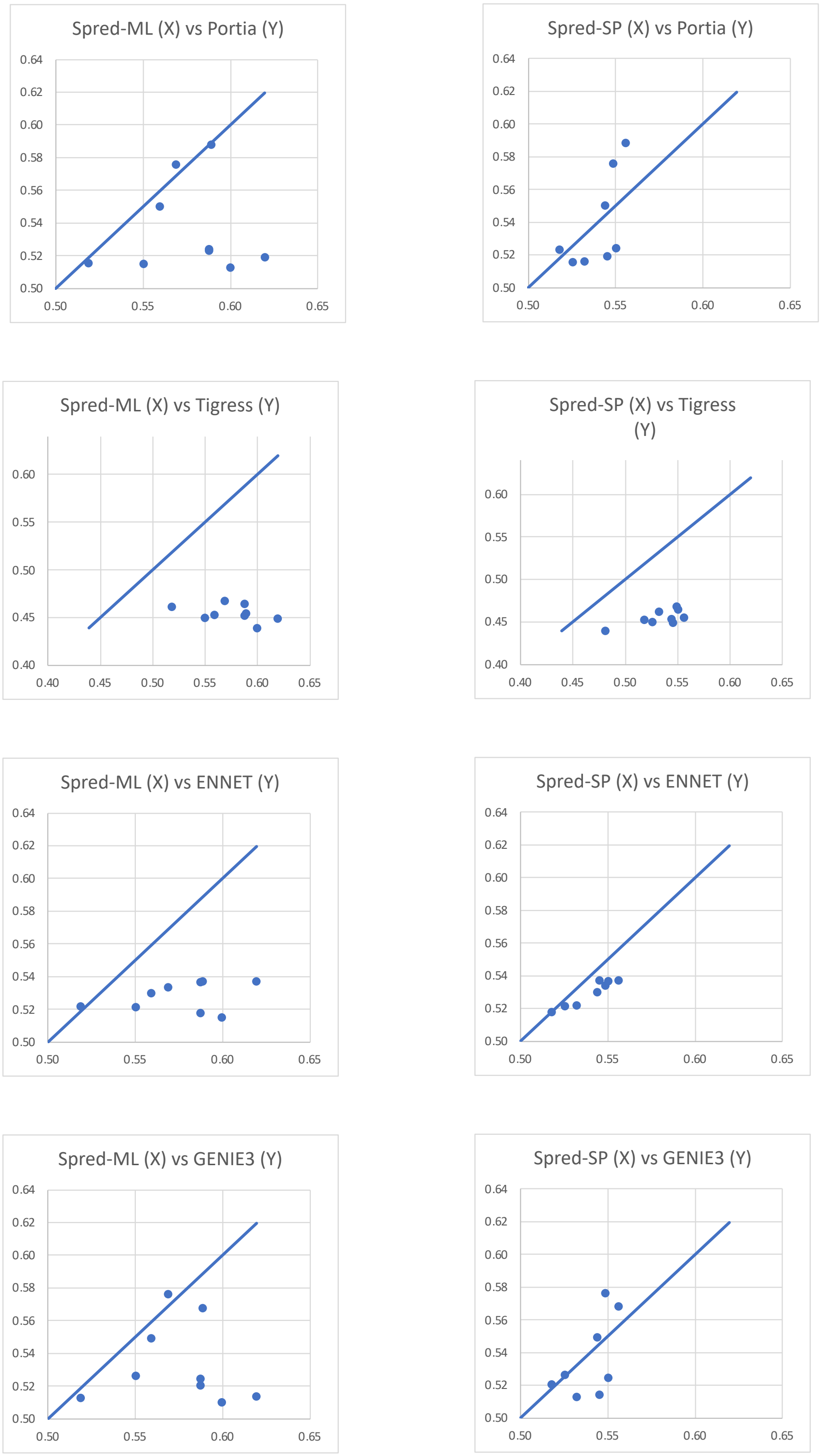

Supplementary figure S7. Head-to-head comparison of SPREd-ML (left) or SPREd-SP (right) versus each comparator (PORTIA, TIGRESS, ENNET, GENIE3) in terms of AUROC on each of the nine benchmarks ("tests"). In each panel, the nine points shown represent the benchmarks, X-axis is the AUROC (averaged over all genes) of a SPREd model and Y-axis is the AUROC of a comparator.

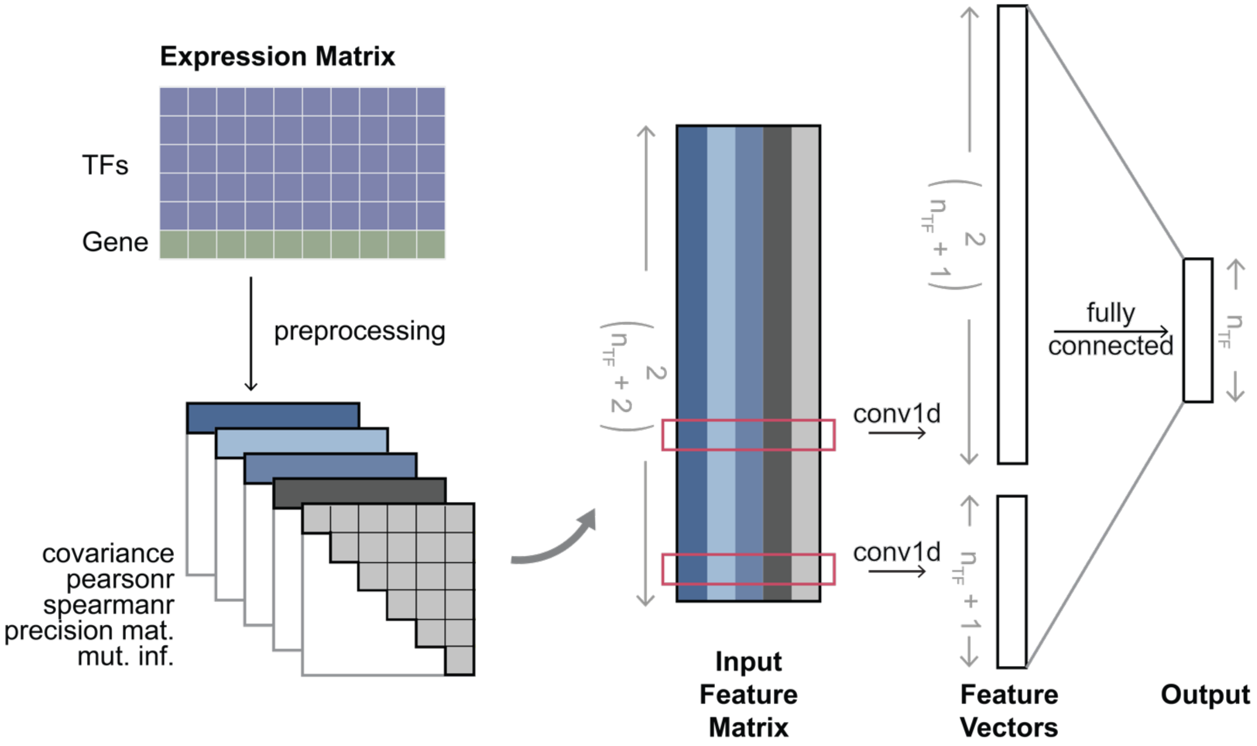

Supplementary figure S8. Architecture of SPREd-ML neural network model. Given an expression matrix whose rows represent n_{TF} TFs and one target gene, the preprocessing step creates five features for each of the $\binom{n_{TF}+1}{2}$ TF-TF pairs and each of the $n_{TF}+1$ TF-target gene pairs. These features include covariance, Pearson correlation, Spearman correlation, mutual information and precision matrix entry corresponding to the TF-TF or TF-target gene pair. The five features of every gene pair then serve as the inputs of a 1D convolutional neural network (CNN). The output layer consists of n_{TF} binary labels indicating if each TF is a regulator of the target gene.

(**A**)

	PORTIA	ENNET	GENIE3	TIGRESS
**100**	6.74E-63	6.29E-45	3.68E-106	2.42E-71
**50**	0.0024	4.34E-179	1.15E-245	2.74E-144
**25**	2.01E-07	1.92E-175	5.13E-161	1.33E-68
**10**	8.89E-15	0	9.90E-108	-

(**B**)

	PORTIA	ENNET	GENIE3	TIGRESS
**100**	1.09E-26	1.07E-05	4.98E-44	4.28E-43
**50**	0.00043	8.32E-97	3.29E-160	9.16E-123
**25**	0.00055	9.97E-108	1.28E-117	8.00E-65
**10**	0.0024	3.95E-250	1.02E-51	-

Supplementary table S1. P-values of paired Wilcoxon test (two-tailed) of difference in AP scores (**A**) and AUROC (**B**) on 5000 genes between SPREd-SP and PORTIA, ENNET, GENIE3, or TIGRESS. Green/red font indicates better/worse SPREd-SP performance.

	n_{TF}	$\mathbf{n_{genes}}$	$\mathbf{n_{cond}}$	$\mathbf{n_{targets}}$
**Network1**	195	1643	805	1387
**Network2**	99	2810	160	367
**Network3**	334	4511	805	922
**Network4**	333	5950	536	1798

Supplementary table S2. Summaries of different networks in the DREAM5 benchmark, including the number of TFs (n_{TF}), the number of total genes (n_{genes}), the number of conditions (n_{cond}), and the number of target genes ($n_{targets}$) for each network. Source: https://www.synapse.org/#!Synapse:syn2787209/wiki/70350

(**A**)

	SPREd-SP	PORTIA	GENIE3	ENNET
**Network1**	0.31	0.52	**0.58**	0.035
**Network2**	**0.16**	0.12	0.13	0.055
**Network3**	**0.20**	0.17	0.16	0.017
**Network4**	**0.053**	0.039	0.034	0.017

(**B**)

	SPREd-SP	PORTIA	GENIE3	ENNET
**Network1**	0.71	0.85	**0.87**	0.50
**Network2**	0.61	0.61	**0.63**	0.53
**Network3**	0.67	0.67	**0.71**	0.49
**Network4**	0.54	0.54	**0.55**	0.50

Supplementary table S3. Performance evaluation on different networks from the DREAM5 benchmark. We compared the performance of different methods in terms of Average Precision (AP) (**A**) and AUROC (**B**). TIGRESS was not included here since it has a runtime issue and the runs failed to complete.

(A) Runtime for training

$n_{TF} \times n_{genes}$	**Time (s) per epoch**
2500000	378.45
1500000	222.87
500000	76.741

(B) Runtime for testing

$n_{TF} \times n_{genes}$	**Time (s)**
500000	558.43
100000	109.45
50000	54.52

Supplementary table S4. Run-time and scalability of SPREd-SP. (**A**) Run time for training the SPREd model, per epoch, for varying data sizes ($n_{TF} \times n_{genes}$). A full training typically spans 50 epochs. (**B**) Run time for applying a trained SPREd model to infer GRN for data sets of varying sizes.

	AP	AUROC
**SPREd**	0.35	0.76
**MLP**	0.26	0.70
**p-value**	0	1.23e-310

Supplementary table S5. Comparison of AP score and AUROC between SPREd-SP CNN architecture and simple 2-layer MLP. Pair-wise Wilcoxon p-values comparing SPREd and MLP model are listed at the bottom.

	MacIsaac2	Yeastract Type2	Yeastract Count3
**NatVar**	1.54	1.74	2.14
**KO**	1.19	2.28	1.66
**Stress**	2.39	1.54	1.25

Supplementary table S6. Mean of “fold change AP” of SPREd-ML over all genes, for each of the nine benchmarks. Fold change AP refers to the observed AP divided by the random baseline AP for a gene.
